# Healthcare claims-based Lyme disease case-finding algorithms in the United States: A systematic literature review

**DOI:** 10.1371/journal.pone.0276299

**Published:** 2022-10-27

**Authors:** Young Hee Nam, Sarah J. Willis, Aaron B. Mendelsohn, Susan Forrow, Bradford D. Gessner, James H. Stark, Jeffrey S. Brown, Sarah Pugh

**Affiliations:** 1 Department of Population Medicine, Harvard Medical School and Harvard Pilgrim Health Care Institute, Boston, Massachusetts, United States of America; 2 Vaccines Medical and Scientific Affairs, Pfizer, Inc., Collegeville, Pennsylvania, United States of America; Federal University of Mato Grosso do Sul, BRAZIL

## Abstract

**Background and objective:**

Lyme disease (LD) is the fifth most commonly reported notifiable infectious disease in the United States (US) with approximately 35,000 cases reported in 2019 via public health surveillance. However, healthcare claims-based studies estimate that the number of LD cases is >10 times larger than reported through surveillance. To assess the burden of LD using healthcare claims data and the effectiveness of interventions for LD prevention and treatment, it is important to use validated well-performing LD case-finding algorithms (“LD algorithms”). We conducted a systematic literature review to identify LD algorithms used with US healthcare claims data and their validation status.

**Methods:**

We searched PubMed and Embase for articles published in English since January 1, 2000 (search date: February 20, 2021), using the following search terms: (1) “Lyme disease”; and (2) “claim*” or “administrative* data”; and (3) “United States” or “the US*”. We then reviewed the titles, abstracts, full texts, and bibliographies of the articles to select eligible articles, i.e., those describing LD algorithms used with US healthcare claims data.

**Results:**

We identified 15 eligible articles. Of these, seven studies used LD algorithms with LD diagnosis codes only, four studies used LD diagnosis codes and antibiotic dispensing records, and the remaining four studies used serologic test order codes in combination with LD diagnosis codes and antibiotics records. Only one of the studies that provided data on algorithm performance: sensitivity 50% and positive predictive value 5%, and this was based on Lyme disease diagnosis code only.

**Conclusions:**

US claims-based LD case-finding algorithms have used diverse strategies. Only one algorithm was validated, and its performance was poor. Further studies are warranted to assess performance for different algorithm designs and inform efforts to better assess the true burden of LD.

## Introduction

Lyme disease (LD) (also known as Lyme borreliosis), caused by the *Borrelia* bacterium transmitted to humans by ticks in the genus *Ixodes*, is the fifth most commonly reported notifiable infectious disease [1] and the most frequently reported vector-borne disease [2] in the United States (US). According to the most recent surveillance reports, approximately 35,000 LD cases were reported to the US Centers for Disease Control and Prevention (CDC) via public health surveillance in 2019 [1]. However, the true burden of LD remains unclear. Because public health surveillance is a passive reporting system, underreporting of true cases exists. Healthcare claims data are another source that can provide information on LD patients. A recent study conducted by CDC researchers using claims data estimated that approximately 476,000 patients were diagnosed and treated for LD in the US annually during 2010–2018 [3], suggesting a remarkably larger clinical and societal burden of LD than estimated via surveillance data. The reliability of claims-based LD estimates, however, depends on the performance of the case-finding algorithms among other factors. To assess the true burden of LD and the effectiveness of the interventions to prevent and treat LD, it is important to use validated well-performing LD case-finding algorithms. We conducted a systematic literature review to identify LD case-finding algorithms used with US healthcare claims data and their validation status.

## Methods

### Protocol

We developed a protocol (described below; not registered on a public website), using the guidelines of the Preferred Reporting Items for Systematic Reviews and Meta-Analyses (PRISMA) Statement [4] and making adjustments needed for the items that were not applicable to our study (e.g., for the items applicable to the literature review to assess the outcomes of clinical trials or interventions). Our PRISMA checklist and database search strategies are presented in **S1 and S2 Tables**, respectively.

### Information sources, literature eligibility, search, and selection

**Fig 1:**
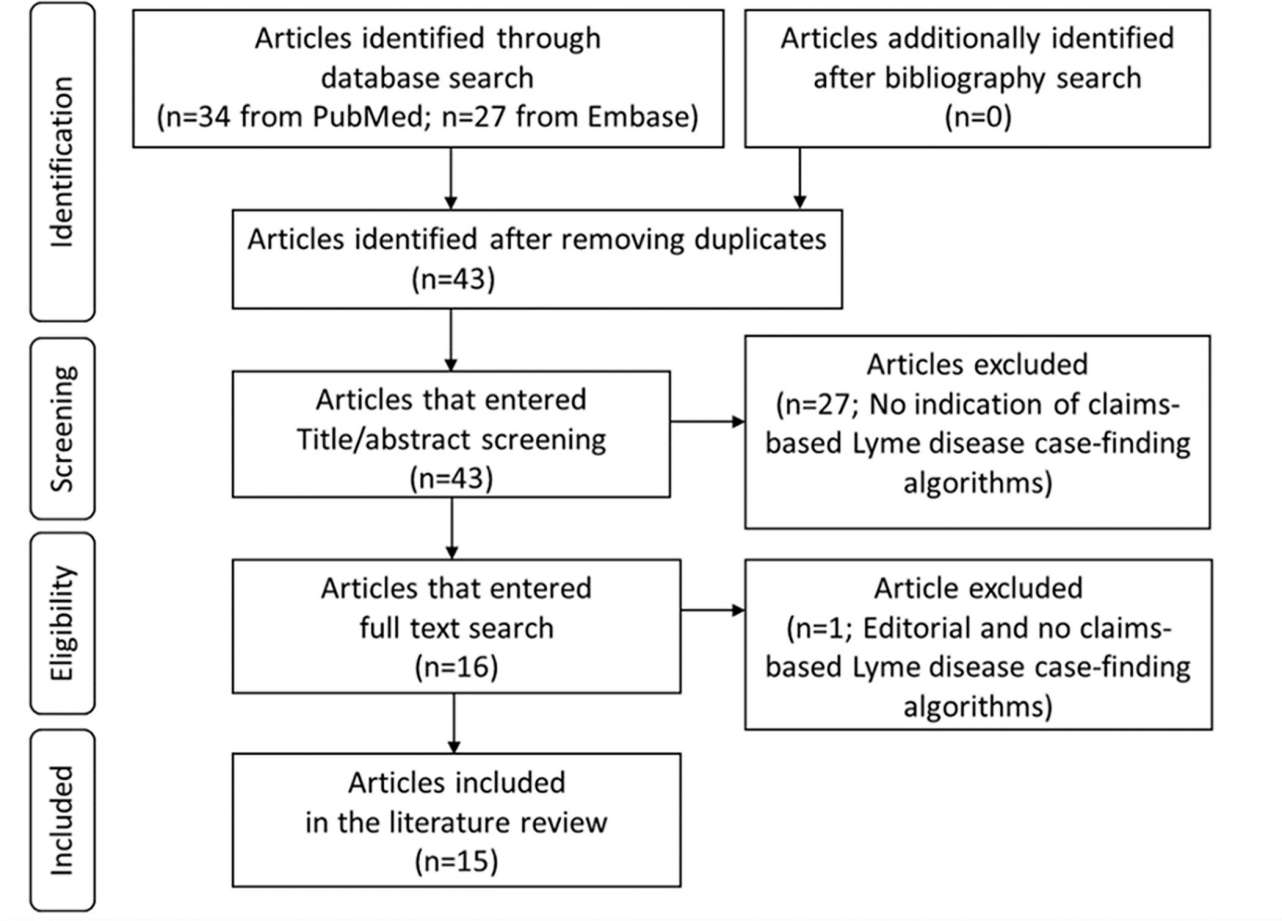
Search and selection of articles for the literature review on the US claims-based Lyme disease case-finding algorithms published in English since 2000. Reference for the flow diagram: Preferred Reporting Items for Systematic Reviews and Meta-Analyses (PRISMA) Statement (https://doi.org/10.1371/journal.pone.0210242.g001).

We searched PubMed and Embase databases and identified articles published in English since January 1, 2000 (search date: February 20, 2021), using the following search terms (not restricting to article titles or abstracts): (1) “Lyme disease”; and (2) “claim*” or “administrative* data”; and (3) “United States” or “the US*”. We then reviewed the titles, abstracts, full texts, and bibliographies of the articles to select eligible articles, i.e., those describing LD case-finding algorithms used with US administrative healthcare claims data. **Fig 1** shows the process of the search and selection of the articles.

### Data items, data collection, and review

We then further reviewed the selected articles and extracted information on the LD case-finding algorithms, including the following items: study populations; sources and years of the claims data used; elements of the LD case-finding algorithms (e.g., diagnosis codes, dispensing records of medications, serologic test order codes); and algorithm performance if the algorithm was validated. As our literature review was not designed to assess or compare the results of clinical trials, interventions, or any outcome measurements from inferential statistical analyses, assessment of risk of bias in individual studies and across studies (renamed from “quality assessment” in the Quality of Reporting of Meta-Analyses [QUOROM] Statement [5]) was not applicable to our study. We summarized the extracted information on the LD case-finding algorithms.

## Results

From the database search using the prespecified search strategies, we identified 34 articles from PubMed and 27 articles from Embase. After removing duplicates, 43 articles remained. Of these, 27 articles were excluded because there was no indication of claims-based Lyme disease case-finding algorithms in their titles or abstracts. Of the remaining 16 articles, one article was excluded based upon a full text review. Therefore, 15 eligible articles were included in this literature review (**Fig 1**). The 43 articles initially identified through the database search are listed in **S3 Table** with reasons for exclusion for the excluded articles (n = 28).

**Table 1** presents summarized information of the LD case-finding algorithms identified in the 15 articles. Seven studies [7–11, 14, 15] used algorithms identifying LD cases from claims data using LD diagnosis codes only (International Classification of Diseases, 9th Revision [ICD-9], 088.81; ICD-10, A69.2, A69.2x). Four studies [3, 12, 13, 16] used LD diagnosis codes and antibiotic dispensing records, and the remaining four studies [6, 17–19] used serologic test order codes (Current Procedural Terminology [CPT], 86617, 86618) in conjunction with LD diagnosis codes and antibiotic dispensing records. Three studies [3, 13, 16] used different algorithms for inpatient and outpatient settings. Only one study (in Tennessee, a low-incidence state for LD) [8] provided algorithm validation results from a medical records review. This review was conducted using medical records from a commercial health insurance plan and included individuals who were reported as LD cases to the Tennessee Department of Health (TDH) and subset of individuals who were not reported to the TDH. That algorithm used a LD diagnosis code alone; its sensitivity was 50% and positive predictive value was 5%. Quantitative or qualitative comparisons of the reported performance of different LD algorithms were not conducted in any of these studies.

**Table 1:** Lyme disease case-finding algorithms used with the US administrative healthcare claims data identified in the articles published in English since 2000.

Reference	Study population	Data source/year	Lyme disease case-finding algorithm				Algorithm validation data
			Description	Diagnosis codes	Antibiotics treatment	Serologic test order codes	
Adrion et al. 2015 [6]	Commercially-insured individuals age <65 years	IMS Health LifeLink Health Plan Claims Database, 2006–2010	• LD diagnosis code (ICD-9, 088.81) and antibiotic treatment within 30 days of diagnosis; or • LD test order code (CPT, 86618) and antibiotic treatment within 30 days of test; or • LD test order code and LD diagnosis code and antibiotic treatment within 30 days of test (Antibiotics: amoxicillin, amoxicillin-clavulanate, cefotaxime, ceftriaxone, cefuroxime, doxycycline, erythromycin, penicillin, tetracycline)	✓	✓	✓	NA
Beach et al. 2020 [7]	Children captured in the Pediatric Health Information System (PHIS) database	PHIS database; all ED and inpatient encounters at participating children’s hospitals between January 1, 2007 and December 31, 2013	LD diagnosis code (ICD-9, 088.81)	✓			NA
Clayton et al. 2015 [8]	Commercially-insured individuals	Blue Cross Blue Shield of Tennessee claims data, January 2011-June 2013	≥3 primary or secondary codes for LD diagnosis (ICD-9, 088.81)	✓			Sensitivity: 50% PPV: 5%
Goodlet et al. 2018 [9]	Commercially-insured individuals	Truven Health Market Scan Commercial Claims and Encounters Database, 2013–2015	LD diagnosis code, ICD-9 (088.81) or ICD-10 (A69.2)	✓			NA
Jones et al. 2012 [10]	Tennessee residents	Administrative medical claims data from Tennessee’s largest managed care organization, January 1, 2000-December 31, 2009	A primary or secondary LD diagnosis code (ICD-9, 088.81)	✓			NA
Jones et al. 2013 [11]	Tennessee residents	Administrative medical claims data from BlueCross BlueShield of Tennessee from 2000–2009	Primary or secondary diagnosis code (ICD-9, 088.81) and at least three separate corroborating events	✓			NA
Kugeler et al. 2021 [3]	Commercially-insured individuals	IBM Watson Health MarketScan Commercial Claims and Encounters Databases, 2010–2018	• Nelson et al. 2015^4^ algorithm (diagnosis code, antimicrobial prescription) • For diagnosis code, ICD-10-CM code (A69.2x) was additionally used	✓	✓		NA
Montejano 2014 [12]	Commercially-insured individuals	Truven Health MarketScan Treatment Pathways data, 2010–2012	LD diagnosis (ICD-9, 088.81) and ≥14 days’ supply of first-line oral antibiotics (amoxicillin, doxycycline, cefuroxime axetil)	✓	✓		NA
Nelson et al. 2015 [13]	Commercially-insured individuals, age <65 years	Truven Health MarketScan Commercial Claims and Encounters Database from 50 states, 2005–2010	• Inpatient event (measured on the date of admission): hospital admission with the ICD-9-CM diagnosis code (088.81) (1) as the principal diagnosis or (2) secondary diagnosis plus diagnosis consistent with an established manifestation of LD or plausible co-infection ○ ICD-9-CM diagnosis codes for established manifestations of LD: meningitis (320.7); meningitis, unspecified (322.9); meningitis due to unspecified bacterium (320.9); acute pericarditis (420.xx); myocarditis (422.xx, 429.xx); conduction disorders (426.xx); arthropathy (716.9x); arthropathy associated with infections (711.xx); pain in joint (719.4x); joint effusion (719.0x); facial weakness (438.83); injury to facial nerve (951.4) or other specified cranial nerves (951.8); neuralgia, neuritis, and radiculitis unspecified (729.2); nerve lesions (353.xx, 354.xx, 355.xx); acute infective polyneuritis (357.0); polyneuropathy in other diseases classified elsewhere (357.4); unspecified inflammatory and toxic neuropathies (357.9) ○ ICD-9-CM codes for co-infection: babesiosis (088.82); anaplasmosis/Ehrlichiosis (082.4x) • Outpatient event: (1) any outpatient or ED visit with ICD-9-CM diagnosis code (088.81) and (2) a prescription filled for an antimicrobial drug recommended by the IDSA for LD treatment + three additional antimicrobial drugs (*) (because they were closely related to a recommended antimicrobial drug or were a known historical treatment that some practitioners might still prescribe); only prescriptions of at least seven days’ duration and filled ±30 days from the visit date (Antimicrobial drugs: amoxicillin; amoxicillin/clavulanic acid (*); azithromycin or azithromycin dihydrate; doxycycline (all forms); cefotaxime sodium; ceftriaxone sodium; cefuroxime axetil; clarithromycin; erythromycin—all forms except lactobionate [IV], gluceptate [IV], thiocyanate (not available in the US), and ethylsuccinate/sulfisoxazole; minocycline hydrochloride (*); penicillin G (benzathine, procaine, or potassium), tetracycline hydrochloride (*). ‘*’ indicates three additional antimicrobial drugs.)	✓	✓		NA
Rebman et al. 2018 [14]	Maryland residents, age <65 years	Data from Priority Partners (a large Medicaid managed-care organization in Maryland) from July 2004-June 2011	LD diagnosis code, ICD-9-CM (088.81)	✓			NA
Schwartz et al. 2020 [15]	Commercially-insured individuals, age <65 years	Inpatient records from the Truven Health Analytics MarketScan Commercial Claims and Encounters Databases, 2005–2014	• A principal diagnosis code for LD (ICD-9-CM, 088.81); or • A principal diagnosis code for a known LD manifestation or co-infection of LD (*) + a secondary diagnosis code for LD* ICD-9-CM diagnosis codes for known manifestations or co-infection of LD: • Facial palsy: 351.0, 351.8, 351.9, 352.6, 352.9, 781.94, 951.4, 951.8, 951.9 • Lyme carditis: 420.xx, 422.xx, 426.0, 426.1x, 426.2, 426.3, 426.4, 426.5x, 426.6, 426.89, 426.9, 427.81, 429.0 • Meningitis: 320.7, 320.82, 320.89, 320.9,322.0, 322.9 • Arthritis: 711.0x, 711.4x, 711.8x, 711.9x, 716.6x, 719.0x • Co-infection: 088.82, 082.4x	✓			NA
Schwartz et al. 2021 [16]	Commercially-insured individuals, age <65 years	IBM Watson Health MarketScan Commercial Claims and Encounters Databases, 2010–2018	• Inpatient cases: (1) a principal diagnosis code for LD (ICD-9-CM, 088.81; ICD-10-CM, A69.2) or (2) a principal diagnosis code of a documented objective clinical manifestation of LD or a tickborne disease transmitted by the same vector (e.g., babesiosis) and a secondary diagnosis code for LD in the same record • Outpatient cases: LD diagnosis code and a prescription for ≥7 days of treatment with an antimicrobial drug appropriate for LD and filled within 30 days before or after the encounter date	✓	✓		NA
Tseng et al. 2015a [17]	Residents in 13 states with high-prevalence of LD (CT, DE, ME, MD, MA, MN, NH, NJ, NY, PA, VT, VA, WI)	Claims data from a nationwide health insurance plan during 2004–2006 and 2010–2012	• At least one LD diagnosis code (ICD-9, 088.81); and • At least one CPT code (86617 or 86618) for serologic testing for *B*. *burgdorferi* within 90 days before or after the first LD condition era (merged into a condition era if the interval between two claims with the LD diagnosis code <30 days, i.e., a 30-day persistence window was applied); and • Antibiotic treatment ≥14 days with antibiotics recommended by IDSA (doxycycline, amoxicillin, cefuroxime axetil, ceftriaxone, cefotaxime, penicillin G, and azithromycin, clarithromycin, erythromycin for adult patients intolerant of amoxicillin, doxycycline, and cefuroxime axetil) within 30 days before or after the first LD condition era; a 30-day persistence window was applied	✓	✓	✓	NA
Tseng et al. 2015b [18]	Residents in 14 states with high-prevalence of LD (CT, DE, ME, MD, MA, MN, NH, NJ, NY, PA, RI, VT, VA, WI)	Claims data from a nationwide employer-provided health insurance plan during 2004–2006 and 2010–2012	• ≥1 LD diagnostic code (ICD-9, 088.81) in the principal diagnosis field between January 1, 2004 and December 31, 2006 or between January 1, 2010 and December 31, 2012; and • ≥1 serologic test order for *B*. *burgdorferi* (CPT, 86618 or 86617) within 90 days before or after the LD condition era; and • ≥1 order for treatment defined as ≥2-week course of one of the antibiotics recommended for the treatment of LD by the IDSA (doxycycline, amoxicillin, cefuroxime axetil, ceftriaxone, cefotaxime, penicillin G, and azithromycin, clarithromycin, and erythromycin for adult patients intolerant of amoxicillin, doxycycline, and cefuroxime axetil) that began within 30 days before or after any LD condition era. For the extended use of antibiotics in patients evaluated for LD, the length of antibiotic treatment was required to be ≥5 weeks.	✓	✓	✓	NA
Tseng et al. 2017 [19]	Residents in 14 states with high-prevalence of LD (CT, DE, ME, MD, MA, MN, NH, NJ, NY, PA, RI, VT, VA, WI)	Claims data from a nationwide, private health insurance plan from July 2010 to December 2012	• PLDSA: ≥1 LD diagnosis code (ICD-9, 088.81), ≥1 LD serologic testing order code (CPT, 86617 or 86618), and antibiotic therapy for ≥5 weeks (defined in Tseng 2015a^18^) • PLDEA: ≥1 LD diagnosis code (ICD-9, 088.81), ≥1 LD serologic testing order code (CPT, 86617 or 86618), and antibiotic therapy for 2–5 weeks	✓	✓	✓	NA

CPT: Current Procedural Terminology. ED: Emergency department. ICD-9-CM: International Classification of Diseases, 9th revision, Clinical Modification. ICD-10-CM: International Classification of Diseases, 10th revision, Clinical Modification. IDSA: Infectious Diseases Society of America. LD: Lyme disease. NA: Not available. PLDEA: Patients evaluated for Lyme disease and given extended antibiotic therapy. PLDSA: Patients evaluated for Lyme disease and given standard antibiotic therapy.

## Discussion

We identified and reviewed 15 articles describing LD case-finding algorithms used with US healthcare claims data. Of these, seven studies used algorithms with LD diagnosis codes only, and the other studies used algorithms with combinations of a LD diagnosis code(s), dispensing of antibiotic medications, and/or a serologic test order code(s). Only one of the studies provided results from algorithm validation [8], which showed that their LD diagnosis code-only algorithm identified only a half of true LD cases, and a large proportion (95%) of the cases identified in the claims data were false positives. The poor performance of this algorithm might be associated with a variety of clinical manifestations of LD, which makes diagnosing LD challenging, a potentially less sophisticated case-finding algorithm, and the accuracy of the claims data, which might not have been sufficiently high for LD case identification. The performance of LD case-finding algorithms might vary depending upon not just the level of sophistication of the algorithms themselves but also the accuracy of LD diagnosis by physicians and of coding in medical records and claims data, as well as geographic regions with varying LD incidences and where risk of a tick bite may vary. Previous studies documented that considerable proportions of LD patients reported through public surveillance lacked an ICD diagnosis code in their medical records [13, 20], and false positive LD cases were also common in medical records [21]. This suggests inaccuracies in medical records, and thus possibly claims data as well.

Six of the 15 articles compared LD incidence estimated using their LD case-finding algorithms with LD incidence from surveillance data [3, 11, 13, 14, 16, 17]. In these studies, claims-based incidence rates were higher than those from surveillance data. For example, one study that used an LD diagnosis code-only algorithm reported that claims-based LD incidence rate in Tennessee during 2000–2009 was >7 times the rate based on surveillance data [11]. Another study that used an algorithm with a combination of diagnosis codes and antibiotic dispensing records reported that claims-based LD incidence rate in the US during 2005–2010 was >11 times the rate estimated by surveillance data [13]. However, because only one algorithm was validated and there was variability in their study designs (e.g., data sources, inclusion/exclusion criteria of individuals, years under study), it is not feasible to determine which case-finding algorithms have better performance or whether there are any patterns in the incidence rates across different algorithms by comparing these incidence rate estimates. Future studies will need to validate LD case-finding algorithms with different designs and assess their performance.

Our findings provide a detailed description of the elements of each LD algorithm and can serve as a useful tool to aid interpretation across existing claims-based estimates. Further, this study is important to spur future research regarding the reliability of LD case finding using administrative claims data and the need to develop and validate claims-based LD case-finding algorithms. We searched only two databases (PubMed and Embase), and though this may be a limitation of our study, these databases are among the largest for biomedical and public health literature searches.

In summary, we found that diverse LD case-finding algorithms have been used with US claims data. Only one of the algorithms that used LD diagnosis code alone was validated, and it did not perform well. Further studies are warranted to assess algorithm performance for different designs and inform the efforts to better assess the burden of LD.

## Supporting information

S1 Table PRISMA checklist. (PDF)

S2 Table Database search strategies. (PDF)

S3 Table Articles identified through PubMed and Embase search using prespecified search terms for US healthcare claims-based Lyme disease case-finding algorithms published in English since 2000. (PDF)
