# Supplementary material for: Healthcare claims-based Lyme disease case-finding algorithms in the United States: A systematic literature review
Source: PLoS One. 2022 Oct 27;17(10):e0276299. doi: 10.1371/journal.pone.0276299 (PMC9612517; doi:10.1371/journal.pone.0276299)
Supplement: S2 Table — (PDF) [file pone.0276299.s002.pdf]

S2 Table. Database search strategies

PubMed

Operator	Search terms and limits
	Lyme disease
AND	(claim*) OR (administrative* data)
AND	(United States) OR (the US*)
AND	English[la]
AND	2000:2021[dp]

Embase

Operator	Search terms and limits
	‘Lyme disease’/exp
AND	(‘claim*’/exp) OR (‘administrative* data’/exp)
AND	(‘United States’/exp) OR (‘the US*’/exp)
AND	[English]/lim
AND	[2000-2021]/py
