# Supplementary material for: Healthcare claims-based Lyme disease case-finding algorithms in the United States: A systematic literature review
Source: PLoS One. 2022 Oct 27;17(10):e0276299. doi: 10.1371/journal.pone.0276299 (PMC9612517; doi:10.1371/journal.pone.0276299)
Supplement: S3 Table — (PDF) [file pone.0276299.s003.pdf]

S3 Table. Articles identified through PubMed and Embase search using prespecified search terms for US healthcare claims-based Lyme disease case-finding algorithms published in English since 2000

	Reference	Inclusion status for the literature review	Reason for exclusion
1	Adalsteinsson SA, Shriver WG, Hojgaard A, et al. Multiflora rose invasion amplifies prevalence of Lyme disease pathogen, but not necessarily Lyme disease risk. Parasit Vectors. 2018;11(1):54. DOI:10.1186/s13071-018-2623-0	Excluded via title/abstract screening	No indication of claims-based Lyme disease case-finding algorithms
2	Adams AJ, Dering-Anderson A. Pharmacy-based travel health services: state approaches to prescriptive authority. J Phar Technol. 2018;34(4):175-180. DOI: 10.1177/8755122518770465	Excluded via title/abstract screening	No indication of claims-based Lyme disease case-finding algorithms
3	Adrion ER, Aucott J, Lemke KW, Weiner JP. Health care costs, utilization and patterns of care following Lyme disease. PLoS One. 2015;10(2):e0116767. DOI:10.1371/journal.pone.0116767	Included	NA
4	Alaedini A, Lebwohl B, Wormser GP, Green PH, Ludvigsson JF. Borrelia infection and risk of celiac disease. BMC Med. 2017;15(1):169. DOI:10.1186/s12916-017-0926-1	Excluded via title/abstract screening	No indication of claims-based Lyme disease case-finding algorithms
5	Avitabile CM, Harris MA, Chowdhury D. Cardiac magnetic resonance characterizes myocarditis in a 16-year-old female with Lyme disease. World J Pediatr Congenit Heart Surg. 2016;7(3):394-396. DOI:10.1177/2150135115593134	Excluded via title/abstract screening	No indication of claims-based Lyme disease case-finding algorithms (Case report)
6	Baker PJ. The pain of "chronic Lyme disease": moving the discourse in a different direction. FASEB J. 2012;26(1):11-12. DOI:10.1096/fj.11-192898	Excluded via title/abstract screening	No indication of claims-based Lyme disease case-finding algorithms
7	Beach CM, Hart SA, Nowalk A, Feingold B, Kurland K, Arora G. Increasing burden of Lyme carditis in United States children's hospitals. Pediatr Cardiol. 2020;41(2):258-264. DOI:10.1007/s00246-019-02250-9	Included	NA
8	Behrendt CA, Sedrakyan A, Debus ES. Short-term and long-term results of endovascular and open repair of abdominal aortic aneurysms in Germany. Eur J Vasc Endovasc Surg. 2019;58(6):e139-e141. DOI:10.1016/j.ejvs.2019.06.684	Excluded via title/abstract screening	No indication of claims-based Lyme disease case-finding algorithms; also, not US-based data
9	Brock CM, Bañó-Polo M, Garcia-Murria MJ, Mingarro I, Esteve-Gasent M. Characterization of the inner membrane protein BB0173 from Borrelia burgdorferi. BMC Microbiol. 2017;17(1):219. DOI:10.1186/s12866-017-1127-y	Excluded via title/abstract screening	No indication of claims-based Lyme disease case-finding algorithms

	Reference	Inclusion status for the literature review	Reason for exclusion
10	Brownstein JS, Skelly DK, Holford TR, Fish D. Forest fragmentation predicts local scale heterogeneity of Lyme disease risk. Oecologia. 2005;146(3):469-475. DOI:10.1007/s00442-005-0251-9	Excluded via title/abstract screening	No indication of claims-based Lyme disease case-finding algorithms
11	Casjens SR, Di L, Akther S, et al. Primordial origin and diversification of plasmids in Lyme disease agent bacteria. BMC Genomics. 2018;19(1):218. DOI:10.1186/s12864-018-4597-x	Excluded via title/abstract screening	No indication of claims-based Lyme disease case-finding algorithms
12	Clayton JL, Jones SG, Dunn JR, Schaffner W, Jones TF. Enhancing Lyme disease surveillance by using administrative claims data, Tennessee, USA. Emerg Infect Dis. 2015;21(9):1632-1634. DOI:10.3201/eid2109.150344	Included	NA
13	Coughlin JM, Yang T, Rebman AW, et al. Imaging glial activation in patients with post-treatment Lyme disease symptoms: a pilot study using [11C]DPA-713 PET. J Neuroinflammation. 2018;15(1):346. DOI:10.1186/s12974-018-1381-4	Excluded via title/abstract screening	No indication of claims-based Lyme disease case-finding algorithms
14	DeLong A, Hsu M, Kotsoris H. Estimation of cumulative number of post-treatment Lyme disease cases in the US, 2016 and 2020. BMC Public Health. 2019;19(1):352. DOI:10.1186/s12889-019-6681-9	Excluded via title/abstract screening	No indication of claims-based Lyme disease case-finding algorithms
15	Dyer O. US Pentagon is told to investigate claims that Lyme disease is escaped bioweapon from cold war. BMJ. 2019;366:l4784. DOI:10.1136/bmj.l4784	Excluded via title/abstract screening	No indication of claims-based Lyme disease case-finding algorithms (News)
16	Goodlet KJ, Fairman KA. Adverse events associated with antibiotics and intravenous therapies for post-Lyme disease syndrome in a commercially insured sample. Clin Infect Dis. 2018;67(10):1568-1574. DOI:10.1093/cid/ciy329	Included	NA
17	Herrin BH, Beall MJ, Feng X, Papeş M, Little SE. Canine and human infection with *Borrelia burgdorferi* in the New York City metropolitan area. Parasit Vectors. 2018;11(1):187. DOI:10.1186/s13071-018-2774-z	Excluded via title/abstract screening	No indication of claims-based Lyme disease case-finding algorithms
18	Jones SG, Conner W, Song B, Gordon D, Jayakaran A. Comparing spatio-temporal clusters of arthropod-borne infections using administrative medical claims and state reported surveillance data. Spat Spatiotemporal Epidemiol. 2012;3(3):205-213. DOI:10.1016/j.sste.2012.01.001	Included	NA
19	Jones SG, Coulter S, Conner W. Using administrative medical claims data to supplement state disease registry systems for reporting zoonotic infections. J Am Med Inform Assoc. 2013;20(1):193-198. DOI:10.1136/amiajnl-2012-000948	Included	NA

	Reference	Inclusion status for the literature review	Reason for exclusion
20	Kim SH, Weaver SJ, Yang T, Rosen MA. Managing creativity and compliance in the pursuit of patient safety. BMC Health Serv Res. 2019;19(1):116. DOI:10.1186/s12913-019-3935-2	Excluded via title/abstract screening	No indication of claims-based Lyme disease case-finding algorithms
21	Kini V, Ho PM. Interventions to Improve Medication Adherence: A Review. JAMA. 2018;320(23):2461-2473. DOI:10.1001/jama.2018.19271	Excluded via title/abstract screening	No indication of claims-based Lyme disease case-finding algorithms (Review)
22	Kuehn BM. CDC estimates 300,000 US cases of Lyme disease annually. JAMA. 2013;310(11):1110. DOI:10.1001/jama.2013.278331	Excluded via title/abstract screening	No indication of claims-based Lyme disease case-finding algorithms (News)
23	Kugeler KJ, Schwartz AM, Delorey MJ, Mead PS, Hinckley AF. Estimating the frequency of Lyme disease diagnoses, United States, 2010-2018. Emerg Infect Dis. 2021;27(2):616-619. DOI:10.3201/eid2702.202731	Included	NA
24	Laaksonen M, Klemola T, Feuth E, et al. Tick-borne pathogens in Finland: comparison of *Ixodes ricinus* and *I. persulcatus* in sympatric and parapatric areas. Parasit Vectors. 2018;11(1):556. DOI:10.1186/s13071-018-3131-y	Excluded via title/abstract screening	No indication of claims-based Lyme disease case-finding algorithms
25	Lim M, Kirchhof MG. Dermatology-related uses of medical cannabis promoted by dispensaries in Canada, Europe, and the United States. J Cutan Med Surg. 2019;23(2):178-184. DOI:10.1177/1203475418808761	Excluded via title/abstract screening	No indication of claims-based Lyme disease case-finding algorithms
26	Marx GE, Schwartz AM, On C, Hinckley AF. Single-dose doxycycline as Lyme disease post-exposure prophylaxis in a national commercial insurance claims database—the United States, 2014-2017. Open Forum Infect. Dis. 2019;6:S589. DOI:10.1093/ofid/ofz360.1478	Excluded via title/abstract screening	No indication of claims-based Lyme disease case-finding algorithms
27	Montejano LB. Assessing the incidence and treatment of post-treatment Lyme disease syndrome in an administrative claims database. Value Health. 2014;17(3):A267. DOI: 10.1016/j.jval.2014.03.1556	Included	NA
28	Nair KV, Corboy J, Kahler K, et al. Use of diagnostic tests and procedures for disease-modifying therapy users and non-disease-modifying therapy users with multiple sclerosis. Expert Rev Neurother. 2011;11(6):787-798. DOI:10.1586/ern.11.67	Excluded via title/abstract screening	No indication of claims-based Lyme disease case-finding algorithms
29	Nelson CA, Saha S, Kugeler KJ, et al. Incidence of clinician-diagnosed Lyme disease, United States, 2005-2010. Emerg Infect Dis. 2015;21(9):1625-1631. DOI:10.3201/eid2109.150417	Included	NA

	Reference	Inclusion status for the literature review	Reason for exclusion
30	Norris SJ, Barbour AG, Fish D, Diuk-Wasser MA. Analysis of the intergenic sequences provided by Feria-Arroyo et al. does not support the claim of high *Borrelia burgdorferi* tick infection rates in Texas and northeastern Mexico. Parasit Vectors. 2014;7(1):467. DOI:10.1186/s13071-014-0467-9	Excluded via title/abstract screening	No indication of claims-based Lyme disease case-finding algorithms (Letter)
31	Ogden NH, Bouchard C, Badcock J, et al. What is the real number of Lyme disease cases in Canada? BMC Public Health. 2019;19(1):849. DOI:10.1186/s12889-019-7219-x	Excluded via title/abstract screening	No indication of claims-based Lyme disease case-finding algorithms
32	Rebman AW, Wang L, Yang T, et al. Incidence of Lyme disease diagnosis in a Maryland Medicaid population, 2004-2011. Am J Epidemiol. 2018;187(10):2202-2209. DOI:10.1093/aje/kwy133	Included	NA
33	Schwartz AM, Shankar MB, Kugeler KJ, et al. Epidemiology and cost of Lyme disease-related hospitalizations among patients with employer-sponsored health insurance-United States, 2005-2014. Zoonoses Public Health. 2020;67(4):407-415. DOI:10.1111/zph.12699	Included	NA
34	Schwartz AM, Kugeler KJ, Nelson CA, Marx GE, Hinckley AF. Use of commercial claims data for evaluating trends in Lyme disease diagnoses, United States, 2010-2018. Emerg Infect Dis. 2021;27(2):499-507. DOI:10.3201/eid2702.202728	Included	NA
35	Steere AC. Treatment of Lyme arthritis. J Rheumatol. 2019;46(8):871-873. DOI:10.3899/jrheum.190320	Excluded via full text screening	No indication of claims-based Lyme disease case-finding algorithms (Editorial)
36	Taank V, Zhou W, Zhuang X, et al. Characterization of tick organic anion transporting polypeptides (OATPs) upon bacterial and viral infections. Parasit Vectors. 2018;11(1):593. DOI:10.1186/s13071-018-3160-6	Excluded via title/abstract screening	No indication of claims-based Lyme disease case-finding algorithms
37	Thomas N, Rutz HJ, Hook SA, et al. Assessing diagnostic coding practices among a sample of healthcare facilities in Lyme disease endemic areas: Maryland and New York - a brief report. Zoonoses Public Health. 2018;65(2):275-278. DOI:10.1111/zph.12414	Excluded via title/abstract screening	No indication of claims-based Lyme disease case-finding algorithms
38	Tseng YJ, Cami A, Goldmann DA, DeMaria A Jr, Mandl KD. Incidence and patterns of extended-course antibiotic therapy in patients evaluated for Lyme disease. Clin Infect Dis. 2015a;61(10):1536-1542. DOI:10.1093/cid/civ636	Included	NA
39	Tseng YJ, Cami A, Goldmann DA, DeMaria A Jr, Mandl KD. Using nation-wide health insurance claims data to augment	Included	NA

	Reference	Inclusion status for the literature review	Reason for exclusion
	Lyme disease surveillance. Vector Borne Zoonotic Dis. 2015b;15(10):591-596. DOI:10.1089/vbz.2015.1790		
40	Tseng YJ, DeMaria A Jr, Goldmann DA, Mandl KD. Claims-based diagnostic patterns of patients evaluated for Lyme disease and given extended antibiotic therapy. Vector Borne Zoonotic Dis. 2017;17(2):116-122. DOI:10.1089/vbz.2016.1991	Included	NA
41	Wormser GP, Shapiro ED, Strle F. Studies that report unexpected positive blood cultures for Lyme borrelia - are they valid? Diagn Microbiol Infect Dis. 2017;89(3):178-181. DOI:10.1016/j.diagmicrobio.2017.07.009	Excluded via title/abstract screening	No indication of claims-based Lyme disease case-finding algorithms
42	Zolnik CP, Falco RC, Kolokotronis SO, Daniels TJ. No observed effect of landscape fragmentation on pathogen infection prevalence in blacklegged ticks (*Ixodes scapularis*) in the northeastern United States. PLoS One. 2015;10(10):e0139473. DOI:10.1371/journal.pone.0139473	Excluded via title/abstract screening	No indication of claims-based Lyme disease case-finding algorithms
43	HIV-discrimination claim fails for man with two jobs. AIDS Policy Law. 2005;20(4):6.	Excluded via title/abstract screening	No indication of claims-based Lyme disease case-finding algorithms; author list, abstract, full text unavailable

NA: not applicable
